# Ischemia Reperfusion Injury Triggers CXCL13 Release and B-Cell Recruitment After Allogenic Kidney Transplantation

**DOI:** 10.3389/fimmu.2020.01204

**Published:** 2020-08-06

**Authors:** Kirill Kreimann, Mi-Sun Jang, Song Rong, Robert Greite, Sibylle von Vietinghoff, Roland Schmitt, Jan Hinrich Bräsen, Lena Schiffer, Jessica Gerstenberg, Vijith Vijayan, Oliver Dittrich-Breiholz, Li Wang, Christian M. Karsten, Wilfried Gwinner, Hermann Haller, Stephan Immenschuh, Faikah Gueler

**Affiliations:** ^1^Department of Nephrology, Hannover Medical School (MHH), Hannover, Germany; ^2^Nephropathology Unit, Institute of Pathology, Hannover Medical School (MHH), Hannover, Germany; ^3^Institute for Transfusion Medicine, Hannover Medical School (MHH), Hannover, Germany; ^4^Research Core Unit Genomics, Hannover Medical School, Hannover, Germany; ^5^Institute for Systemic Inflammation Research, University of Lübeck, Lübeck, Germany

**Keywords:** ischemia reperfusion injury, delayed graft function, kidney transplantation, B-cell activation, CXCL13

## Abstract

Ischemia reperfusion injury (IRI) is linked with inflammation in kidney transplantation (ktx). The chemokine CXCL13, also known as B lymphocyte chemoattractant, mediates recruitment of B cells within follicles of lymphoid tissues and has recently been identified as a biomarker for acute kidney allograft rejection. The goal of this study was to explore whether IRI contributes to the up-regulation of CXCL13 levels in ktx. It is demonstrated that systemic levels of CXCL13 were increased in mouse models of uni- and bilateral renal IRI, which correlated with the duration of IRI. Moreover, in unilateral renal IRI CXCL13 expression in ischemic kidneys was up-regulated. Immunohistochemical studies revealed infiltration of CD22+ B-cells and, single-cell RNA sequencing analysis a higher number of cells expressing the CXCL13 receptor CXCR5, in ischemic kidneys 7 days post IRI, respectively. The potential relevance of these findings was also evaluated in a mouse model of ktx. Increased levels of serum CXCL13 correlated with the lengths of cold ischemia times and were further enhanced in allogenic compared to isogenic kidney transplants. Taken together, these findings indicate that IRI is associated with increased systemic levels of CXCL13 in renal IRI and ktx.

## Introduction

Ischemia reperfusion injury (IRI) in kidney transplantation (ktx) is linked with inflammation and leukocyte recruitment (1). The extent of IRI depends on donor-related factors and duration of the ischemia time. Short ischemia times (2–3 h) that are encountered in living donor ktx have been associated with better long-term graft survival as compared to deceased donor ktx. In deceased donor renal transplants, median cold ischemia time (CIT) in Germany can be up to 14 to 16 h. Extended CITs (longer than 25 h) have been reported in other countries (2). Prolonged CIT and old donor age increase the risk of developing delayed graft function (DGF) (3). Immunosuppressive agents—typically, a combination of prednisolone, mycophenolatmofetil (MMF), and calcineurin inhibitors (CNI)—are used to inhibit rejection. B-cell inhibitors are only used for pretreatment of recipients in ABO-incompatible living donor ktx to reduce or eliminate blood group antibodies prior to transplantation. Despite these interventions, acute rejection occurs shortly after ktx in 6–12% of deceased allograft recipients and some of these patients have signs of antibody-mediated rejection although donor-specific antibodies are not detectable. Thus, activation of a humoral response may be triggered by IRI itself and immediate production of the chemokine CXCL13 after surgery could be an important mediator of B-cell activation and subsequent antibody- mediated transplant rejection. Recently, we showed that CXCL13 is a biomarker for acute mixed allograft rejection in kidney transplant recipients (4). The major function of CXCL13 is recruitment of CXCR5+ cells (mainly naïve B cells) into lymphoid follicles (5, 6). Moreover, CXCL13 causes resident kidney cells to produce pro-inflammatory cytokines and chemokines (4, 7–9). In liver transplantation, increased numbers of circulating CXCR5+CXCR3-CD4+ T-cells have been shown to correlate with acute transplant rejection (10) and CXCR5+ follicular T-helper cells (Tfh) have been linked to humoral immunity (11). Furthermore, it has been shown that CXCR5+CD8+ T-cells localize to B-cell follicles, express costimulatory proteins, and promote B-cell differentiation along with antibody isotype class switching (12). Here, we demonstrate that renal IRI causes increased levels of systemic CXCL13 with subsequent infiltration of CXCR5+ leukocytes in kidneys not only in models of IRI, but also in that of allogenic ktx.

## Materials and Methods

### Animals

Male C57BL/6 and BalbC mice (11–15 weeks of age, 22–28 g in weight) were housed in a 14/10 h light/dark cycle under conventional conditions. Mice had free access to food (Altromin 1324) and tap water, and were monitored daily for behavior and well-being. All experiments were approved by the local authorities (Lower Saxony State office for Consumer Protection and Food Safety, LAVES: 33.12-42502-04-14/1569 and 33.9-42502-04-07/1396; 09/1637).

### Models of Uni- and Bilateral Kidney IRI

Mice were anesthetized with isoflurane (3–5% induction and 1.5% maintenance). Prior to surgery butorphanol 1 mg/kg s.c was injected for analgesia. Depending on the model, bi- or unilateral renal IRI was induced after their abdomens were opened by renal pedicle clamping using a micro-aneurysm clip for 15–45 min to induce subclinical, transient or progressive acute kidney injury (AKI) (13, 14). Sham surgery was performed in the same manner, but the renal pedicle was not clamped. After surgery mice received metamizol (p.o. 200 mg/kg) in drinking water over 3 consecutive days for postoperative pain control. The animals were monitored daily. At the designated endpoints they were deeply anesthetized and euthanized by total body perfusion with ice cold PBS via the left ventricle. Sham kidneys as well as contralateral kidneys from the unilateral experiments served as control tissues.

### Kidney Transplantation Model

C57BL/6 (H2^b^) mice served as donors and fully mismatched BalbC (H2^d^) mice as recipients for allogenic ktx. For isogenic ktx C57BL/6 mice served as donors as well as recipients. Ktx was performed by a vascular surgeon with >20 year experience in small animal microsurgery. Surgeries were performed after the animals were anesthetized with isoflurane inhalation (35% induction 1.5–2% maintenance) following i.p. injection of butorphanol (2 mg/kg) for analgesia. After surgery mice received metamizol (p.o. 200 mg/kg) in drinking water over 3 consecutive days for postoperative pain control. For graft retrieval the donor's left kidney including the renal vein, renal artery, and ureter were removed en bloc. The recipient was prepared for transplantation by removal of its left kidney. The kidney graft was then transplanted into the lower abdomen of the recipient by end-to-side anastomosis of the renal artery to the aorta and the renal vein to the inferior vena cava (15). The ureter was implanted into the bladder dome (16). Normal renal function was ensured by the remaining native, unaffected right kidney of the recipient. After surgery mice were monitored daily for general health, activity, and well-being. Reasons for study termination included behavioral changes (reduced activity, no food intake) or surgical complications (graft thrombosis, hind limb paralysis, bleeding, urinary leakage, or significant urinary congestion).

### Single Cell RNA sequencing (sc-RNAseq) in Control and IRI kidneys

Kidneys from three male C57BL/6 control mice (no surgery) and three male C57BL/6 mice 7 days after unilateral IRI for 27 min were processed for sc-RNAseq to determine whether CXCR5+ cells were infiltrating the kidney. After kidney retrieval, tissue was sliced and digested by collagenase as described (17). Following red blood cell lysis and dead cell removal using a Miltenyi Dead Cell Removal Kit according to the manufacturer's instructions (Miltenyi Biotec, Bergisch Gladbach, Germany) 10,000 cells per sample were subjected to single cell mRNA-Seq analysis (Chromium Single Cell 3 Reagent Kits v3 User Guide, Document Number CG000183, Rev A; 10x Genomics). Equimolar amounts of libraries were pooled, denatured with NaOH, and finally diluted to 1.8 pM according to the Denature and Dilute Libraries Guide (Document # 15048776 v02; Illumina). 1.3 ml of the denatured pool was sequenced on an Illumina NextSeq 550 sequencer using one third of a High Output Flowcell for 75 cycles per sample (#20024906; Illumina). The proprietary 10x Genomics CellRanger pipeline (v3.0.2) was employed with default parameters. CellRanger was used to build a “pre-mRNA” reference package from reference genome provided by 10x Genomics (Mouse reference dataset 3.0.0; November 19, 2018; mm 10) as described in https://support.10xgenomics.com/single-cell-gene-expression/software/pipelines/latest/advanced/references. Read data were then aligned to the “premRNA” reference package with CellRanger using the aligner STAR to count aligned reads per gene and calculate clustering and summary statistics. Finally, the Loupe Cell Browser from 10x was used to view and revise annotated clusters, based on the implemented tSNE algorithm.

### RNA Extraction and qPCR

Gene-specific primers for CXCL13 (Primer-sequence: fwd-TCT GGA CCA AGA rev-TGA AGA AAG TT), monocyte chemoattractant protein-1 (MCP-1; Mm_Ccl2_1_SG QuantiTect Primer Assay QT00167832), IL_6 (Mm_IL6_1_SG QuantiTect Primer Assay QT00098875), and TNF-α (Mm_TNF_1_SG QuantiTect Primer Assay QT00104006) were used. qPCR was performed as described previously (18). After fixation of renal tissue sections in RNAlater (Ambion) overnight, total RNA was isolated using RNeasy Mini Kit (Qiagen). For quantitative real time PCR (qPCR), 1 μg of DNase-treated total RNA was reverse transcribed using PrimeScript Reverse Transcriptase reagent Kit (Takara) and qPCR was performed with a LightCycler 96 (Roche Diagnostics). For each reaction 10 μl TB Green premix Ex Taq II (Takara), 3 μl DEPC-treated water (Ambion), and 10 pmol of forward and reverse primer were used in each well of a 96-well plate. The PCR reaction was initiated at 95°C (30 s), then 40 cycles followed: 5 s 95°C and 1 min at 60°C. HPRT (Mm_HPRT_1_SG QuantiTect Primer Assay QT00166768) was used as house keeping gene for normalization.

### Cytokine Assays

CXCL13 serum levels were analyzed by ELISA (R&D Systems, MCX130) as described previously (19). Color development was measured by a Tecan spectra ELISA reader (Tecan, Crailsheim, Germany). In addition, a CBA bead assay was used to measure proinflammatory cytokines (IL-6, TNF-a, MCP-1) (BD Biosciences) in blood samples.

### Histology of Renal Tissue After IRI and ktx

Paraffin-embedded renal tissue was serially sectioned (2 μm) and stained with periodic acid Schiff (PAS) reaction. Signs of AKI varied from loss of brush border, an early lesion, to cell detachment from the basement membrane and accumulation of cell debris in the tubular lumen. AKI was characterized using a semi-quantitative grading system: 0 = no acute tubular injury (ATI), 1 = focal ATI with <10% of tubuli in the cortex affected, 2 = moderate ATI with 10–25% of tubuli affected, 3 = severe ATI with 25–50% of tubuli affected, 4 = very severe with >50% of the tubuli affected. Scores for interstitial inflammation and leukocyte infiltration ranged from 0 to 4 correlating with mild, moderate, marked, and severe cell infiltration. Kidney grafts were graded according to the updated Banff classification (20). Analysis was performed without knowledge of the animal group identity by a nephropathologist with >20 years of experience. For further characterization of infiltrating leukocytes, immunostaining was performed with antibodies against Gr-1 (neutrophils) (Biorad, MCA 771G), F4/80 (monocytes/macrophages) (Acris Antibodies, BM4007), CD22 (Southern Biotech, 1580-01), and CD45R (B cells) (eBioscience, 14-0452-82), CD3 (T cells) (Dako, A0452), respectively.

## Results

### IRI Causes Increased Serum Levels and Renal Expression of CXCL13

**Figure 1:**
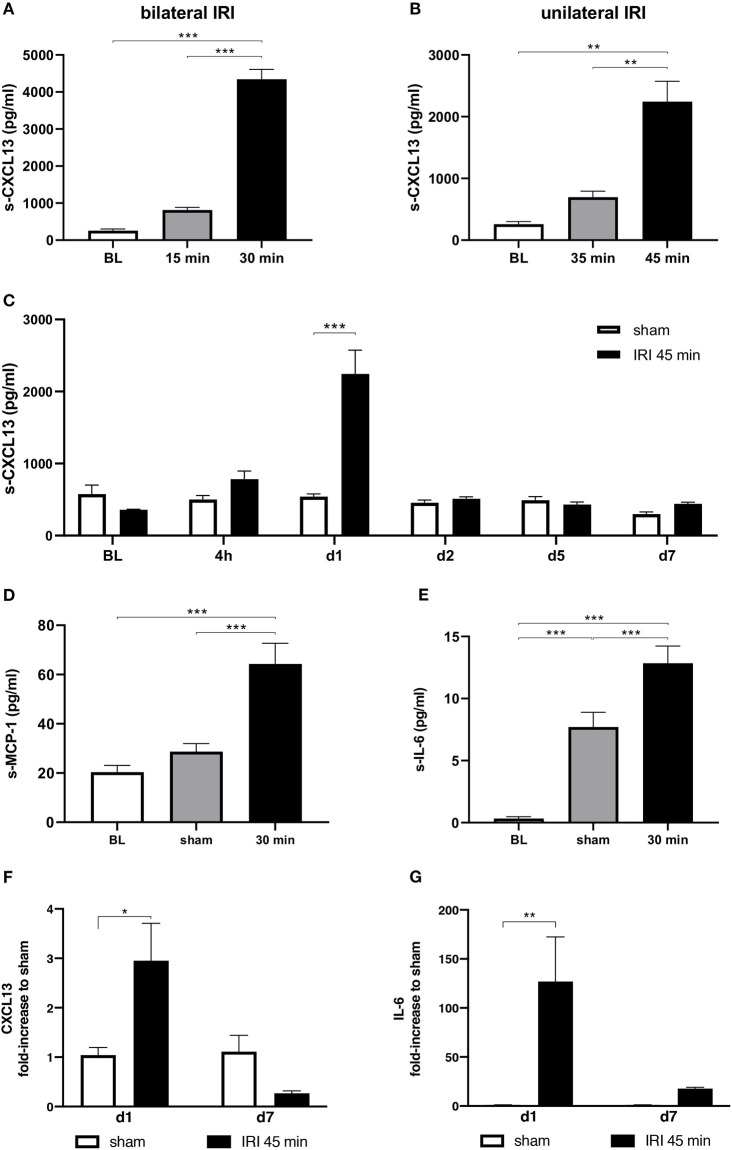
Cytokine levels in serum samples and kidney tissue. Serum levels of pro-inflammatory cytokines were measured in models of uni- and bilateral renal IRI. CXCL13 levels significantly increased within 24 h in 30 min bilateral IRI as compared to 15 min IRI or baseline (BL) levels prior to IRI **(A)**. In unilateral IRI for 35 and 45 min a time-dependent increase of serum CXCL13 levels were observed 24 h after IRI **(B)**. To longitudinally determine the kinetics of CXCL13 release in 45 min unilateral IRI blood samples were taken at the indicated times. A maximum was measured 24 h after IRI **(C)**. MCP-1 **(D)** and IL-6 **(E)** were measured in comparison to sham surgery prior to IRI and 24 h after bilateral IRI. Both markers were significantly increased in comparison to baseline. 24 h after unilateral IRI a significant increase of CXCL13 **(F)** and of IL-6 **(G)** mRNA expression in renal tissue was observed in the 45 min unilateral IRI model (*n* = 6–10 mice per group, one-way ANOVA, *p < 0.05; **p < 0.01, ***p < 0.001). BL, baseline.

Severity of AKI is dependent on duration of renal ischemia times. Here, we compared systemic serum levels of CXCL13 in various models of renal IRI 24 h after surgery. A 15 min ischemia time causes subclinical IRI without elevation of creatinine and blood urea nitrogen (BUN), whereas a 30 min IRI induced these clinical parameters of AKI (Supplementary Figure 1). A pronounced increase of CXCL13 levels in serum was observed in animals that received 30 min compared to 15 min of bilateral IRI (Figure 1A). For comparison, the pro-inflammatory mediators MCP-1 and IL-6 were also significantly elevated after 24 h in the 30 min bilateral IRI model (Figures 1D,E). To allow for longitudinal follow-up without mortality after IRI, a model of unilateral IRI was used, in which only one kidney is clipped with unaltered overall renal function (21). In the unilateral model, 35 min of IRI caused a minor increase, but 45 min of IRI caused a marked increase in the levels of CXCL13 24 h after surgery (Figure 1B). Further time course studies in the 45 min unilateral IRI model revealed that the maximum increase in CXCL13 occurred at day 1 (24 h after IRI), which rapidly returned to basal levels at day 2 and did not increase further until day 7 (Figure 1C). In addition, qPCR analysis of renal samples showed an increase in CXCL13 mRNA expression in ischemic kidneys at day 1 after IRI, but not at day 7 in comparison to renal samples from animals with sham surgery (Figure 1F). Although, a similar time-dependent up-regulation and decline was noted for the pro-inflammatory cytokine IL-6 in ischemic kidneys, the expression levels were still significantly elevated in comparison to sham controls at day 7 after IRI (Figure 1G).

In summary, renal IRI leads to an early increase in systemic serum CXCL13 levels and CXCL13 mRNA expression in ischemic kidneys.

### IRI Causes AKI, Inflammation and Renal Infiltration of B-cells

**Figure 2:**
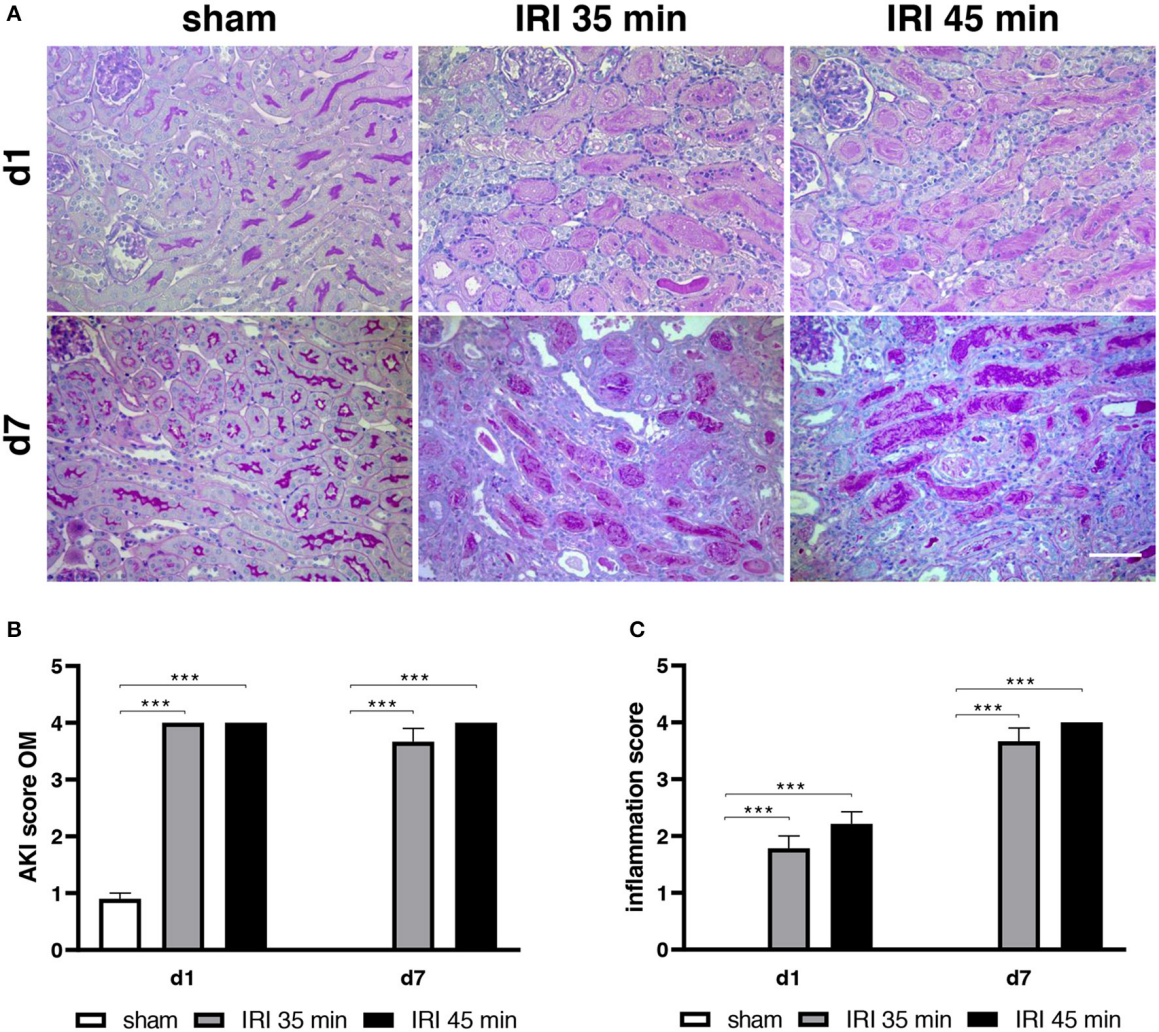
Morphology of kidney tissue. PAS staining revealed significant acute kidney injury (AKI) after 35 and 45 min IRI and almost normal morphology after sham surgery. In **(A)** representative images from the outer medulla are depicted (bar: 100 μm). The respective AKI scoring is shown in **(B)**. The extent of inflammation increased from day 1 until day 7 **(C)**. No differences in the extent of AKI and inflammation between 35 and 45 min IRI were observed by an investigator blinded to animal group assignment (*n* = 6–8 mice per group, two-way ANOVA ***p < 0.001).

**Figure 3:**
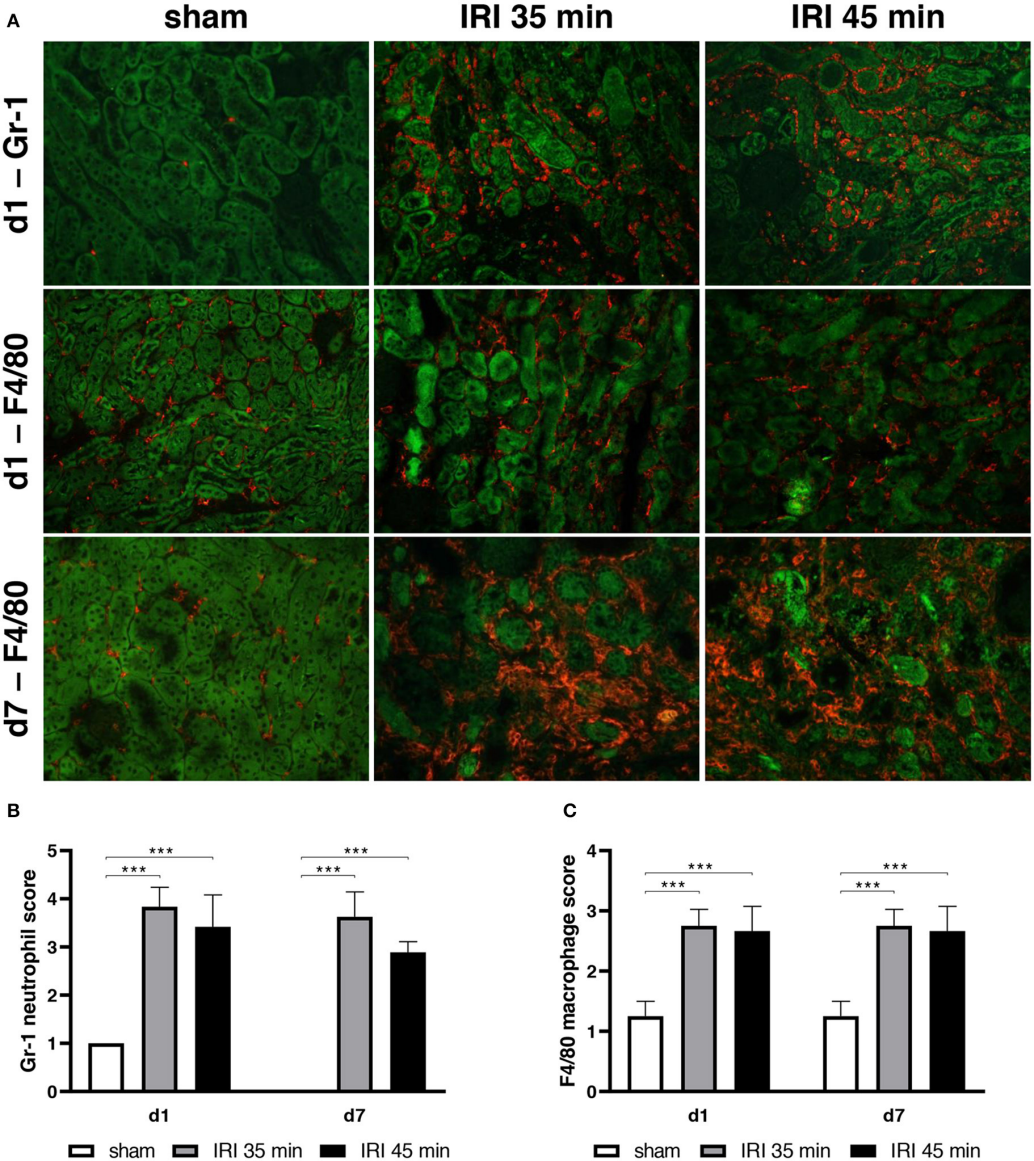
Neutrophil and macrophage infiltration in kidney tissue. Myeloid cell infiltration was determined after unilateral IRI for 35 and 45 min and compared to sham surgery. 24 h after IRI the majority of infiltrating cells were Gr-1+ granulocytes which were mainly detected in the interstitium of the outer medulla (upper row, GR-1 in red, auto-fluorescence of the tubuli in green, **(A,B)**. At day 7 the most prominent cells were F4/80+ **(A,C)**. No differences between 35 and 45 min IRI were observed (bar: 100 μm, *n* = 6–8 mice per group, one-way ANOVA, ***p < 0.001).

**Figure 4:**
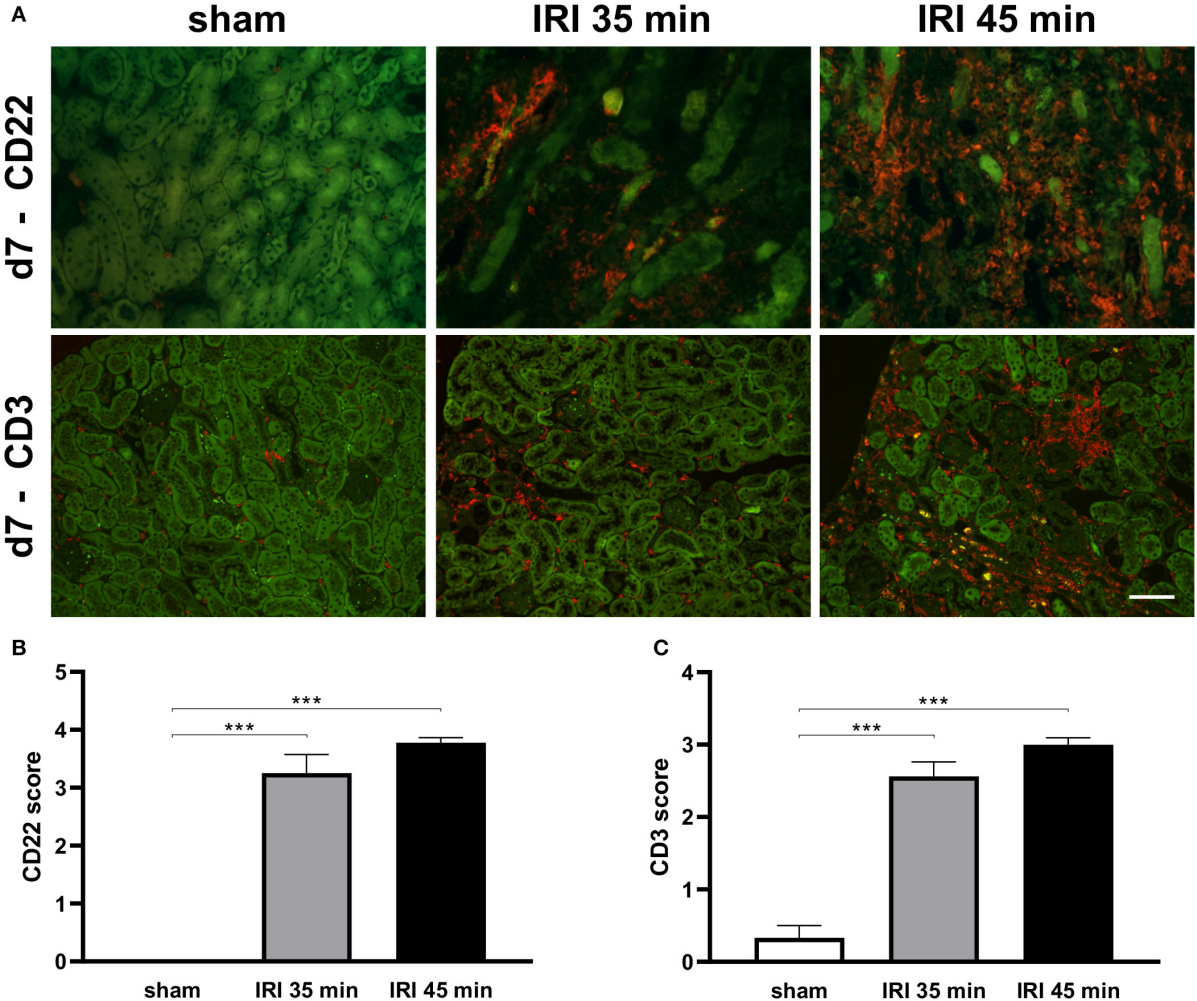
B and T cell infiltration in kidney on day 7 after injury. Infiltration of B and T cells was determined on day 7 after injury. Representative images are shown in **(A)**. Both renal IRI models led to significant increase levels of both cell types as determined by semi-quantitative scoring **(B,C)**. No significant changes between different ischemia times were observed (bar: 100 μm, *n* = 6–8 mice per group, one-way ANOVA, ***p < 0.001).

**Figure 5:**
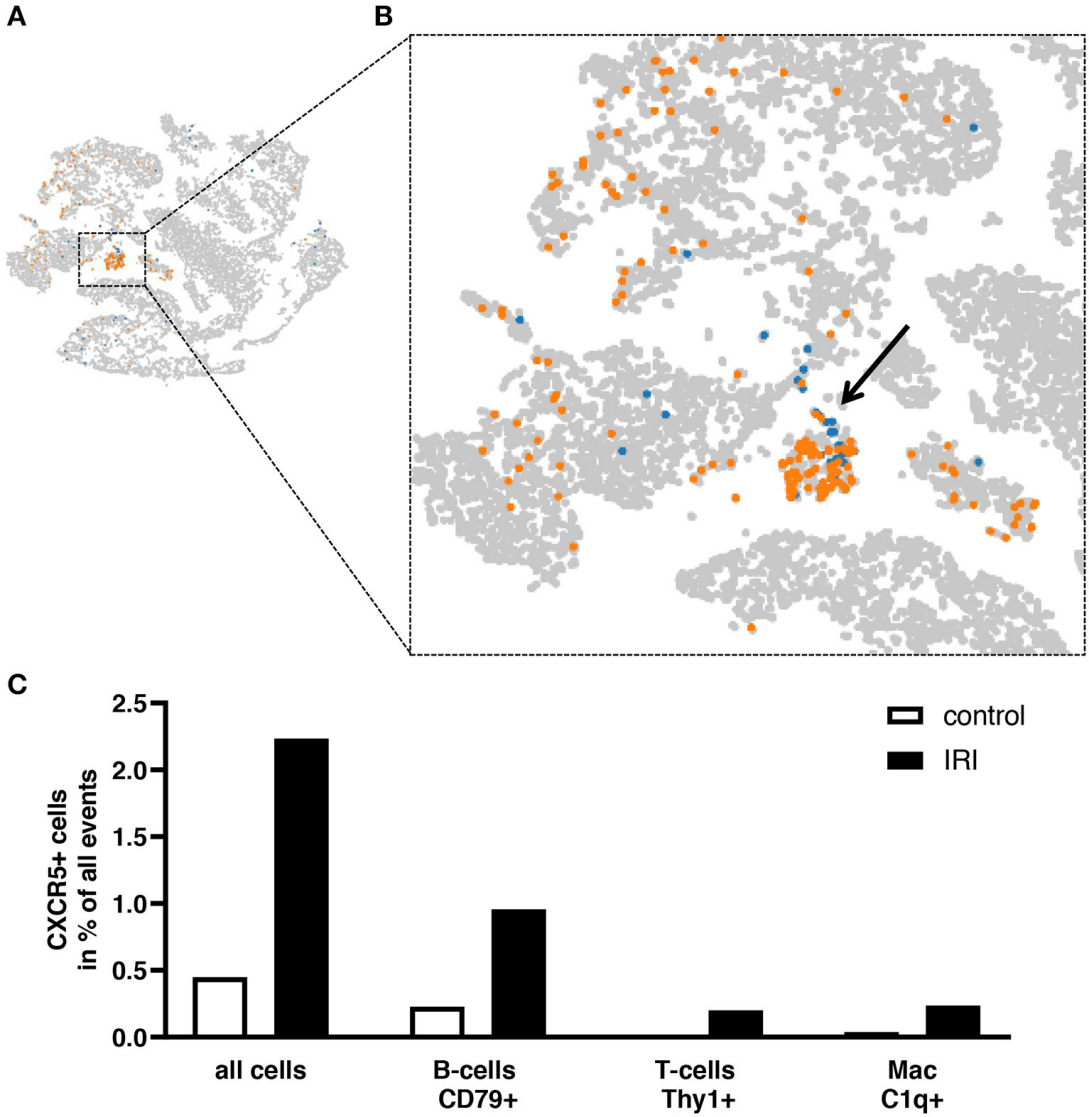
Detection of CXCR5+ cells in kidney tissue by sc-RNAseq. Sc-RNAseq data from three pooled control kidneys were compared to those from three pooled IRI kidneys 7 days after IRI. Expression of CXCR5 was increased after IRI (orange) compared to control (blue). Clusters of CXCR5 positive cells and scattered single cells were identified (**B**, enlarged view of **A**). The highest proportion of CXCR5+ cells were identified amongst CD79+ B-cells, followed by Thy1+ T-cells and C1qc+ macrophages **(C)**.

Histomorphological changes were determined in animals after sham surgery or after 35 min and 45 min of unilateral IRI. In both groups of IRI typical alterations of severe AKI including the presence of apoptotic and necrotic cells at day 1 after surgery were observed and were still detectable at day 7 after IRI. Leukocyte infiltration increased from day 1 to day 7 after 35 and 45 min IRI (Figures 2A–C). As determined by immunohistochemistry the majority of infiltrating cells was GR-1 positive at day 1 (Figures 3A,B), whereas the more prominent cell type was F4/80+ myeloid cells at day 7 (Figures 3A,C). More importantly, at day 7 after IRI an infiltration of CD22+ B-cells was detected in ischemic kidneys, which was almost absent in animals that received sham surgery (Figures 4A,B). In addition, CD3+ T lymphocytes were found in the outer medulla at day 7 (Figures 4A,C). Since CXCL13 recruits cells expressing CXCR5, we performed sc-RNA seq analysis with three pooled control kidneys in comparison to three pooled IRI kidneys to identify CXCR5+ cells (Figure 5). In tSNE analysis IRI kidneys showed a cluster of CXCR5 expressing cells and some scattered single cells (Figures 5A,B). The majority of CXCR5+ cells were of B-cell origin (CD79+) followed by a subset of macrophages (C1qc+) and some T-cells (Thy1+). In control kidneys only few CXCR5+ cells were detected (Figure 5C).

Taken together, the data indicate that renal IRI causes recruitment of B cells to the inflamed tissue.

### CXCL13 Release Depends on Duration of Ischemia Time After Kidney Transplantation

**Figure 6:**
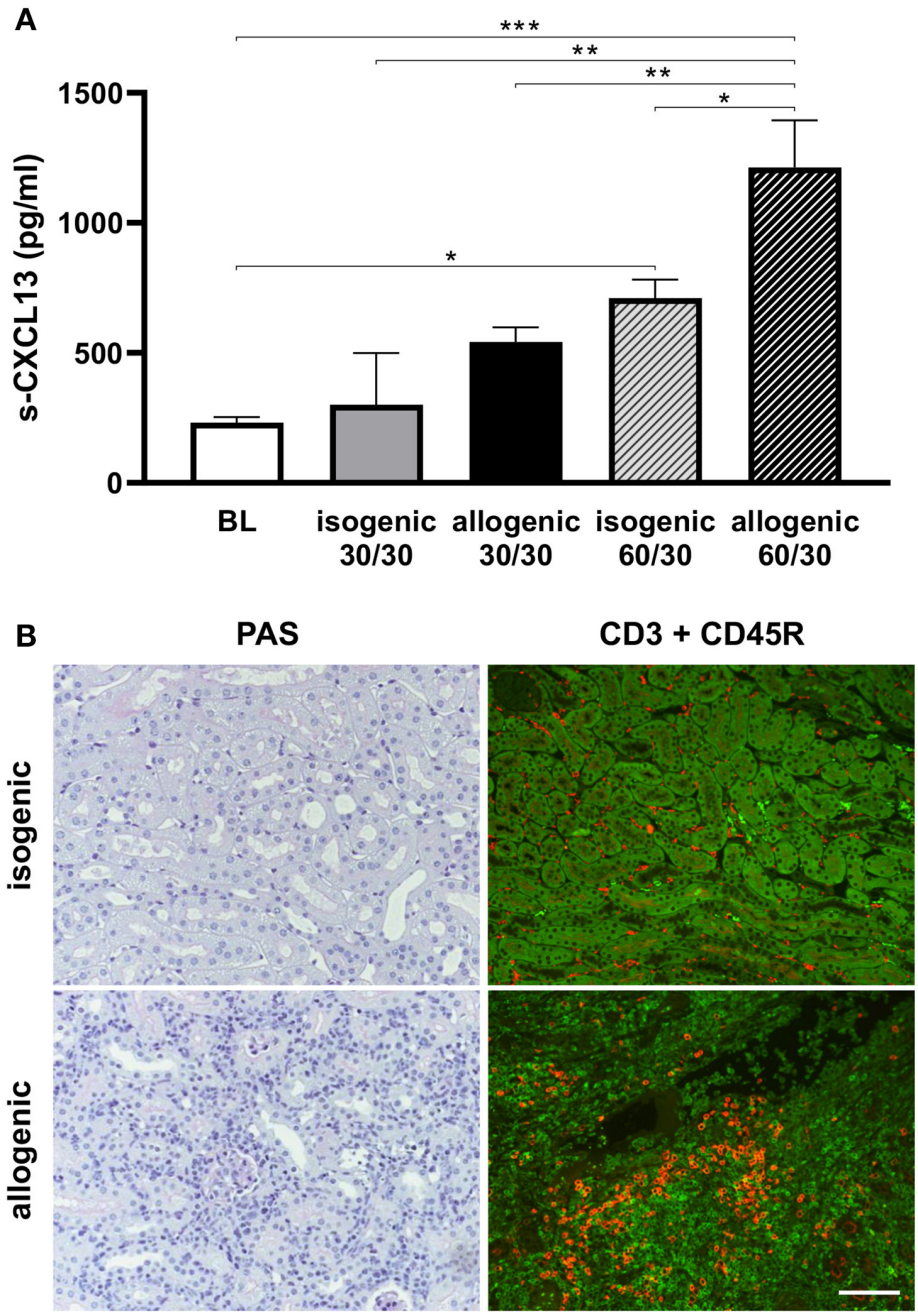
Serum CXCL13 levels in mouse ktx. Post ktx levels of serum CXCL13 at day 1 were significantly increased compared to baseline. A higher increase was observed in longer cold ischemia time (30 vs. 60 min cold ischemia time). Isogenic ktx with prolonged cold ischemia time of 60 min had significantly lower CXCL13 levels compared to allogenic ktx **(A)**. PAS stain at day 7 revealed enhanced cell infiltration in allogenic compared to isogenic ktx **(B)**. Double staining for CD3+ T-lymphocytes (green) and CD45R+ B-cells (red) was performed at day 7. More interstitial CD3+ T-lymphocytes were observed in allografts compared to isografts. Allografts exhibited scattered B-cells in interstitial tissue, but also clusters of CD45R+ cells. Isografts showed only few B cells in the interstitium at day 7 **(B)** (bar: 100 μm, *n* = 6 per group, one-way ANOVA *p < 0.05; **p < 0.01; ***p < 0.001). BL, baseline.

In the following, allogenic ktx [C57BL/6 (H2^b^) on BalbC (H2^d^)] or isogenic ktx was performed with either 30 or 60 min of CIT. Serum levels of CXCL13 were significantly higher in animals that received kidneys with longer CITs. In addition, the increase in levels of systemic serum CXCL13 was amplified in the allogenic as compared to the isogenic transplant setting (Figure 6A). Histopathology revealed inflammation (i.e., Banff 1a rejection) in allografts with enhanced CD45R+ B-cell and CD3+ T lymphocyte infiltration 7 days after transplantation (Figure 6B). To summarize, increased levels of serum CXCL13 levels after ktx are dependent on the duration of CIT. Moreover, systemic levels of CXCL13 and B-cell infiltration into renal grafts post transplantation were higher in the allogenic setting.

## Discussion

This study provides evidence that the chemokine CXCL13, also termed B-lymphocyte chemoattractant, is up-regulated rapidly after renal IRI. Duration of IRI correlated with increased systemic serum CXCL13 levels in both models of uni- and bilateral clamping. Increased levels of CXCL13 in response to IRI were transient and reached a maximum after 24 h of IRI (Figure 1). B-cell infiltrates were detected in the kidney 1 week after IRI (Figure 4). The majority of cells expressing CXCR5 appeared to be of B-cell origin, but some T-cells and macrophages were also CXCR5 positive (Figure 5). Furthermore, in a mouse model of allogenic ktx systemic CXCL13 serum levels correlated with the length of graft ischemia time duration (Figure 6).

It is noteworthy that allogenic ktx caused higher levels of serum CXCL13 levels than isogenic ktx (Figure 6). This finding raises the question of whether the host inflammatory response, which is markedly higher in allogenic ktx may contribute to CXCL13 secretion. A limitation of the current study is that the source of CXCL13 was not identified. A very early event in IRI is rapid complement activation orchestrating the inflammation that follows. Complement activation might also influence the secretion of immune cell-mediated CXCL13 release. Accordingly, recent experimental evidences indicates that activated peritoneal macrophages release CXCL13 via a mechanism that involves the complement anaphylatoxin C5a. Macrophages that are deficient for the C5a receptor C5aR1 showed markedly reduced release of CXCL13. Alternatively, CXCL13 release may also be regulated via Toll-like receptor (TLR)2- and IL-10-dependent mechanisms (22). Since myeloid cells are early drivers of inflammation and the extent of macrophage infiltration is directly linked to the duration of ischemia times and the severity of IRI (21) it is conceivable that infiltrating macrophages after IRI may contribute to CXCL13 production and release. The observation that the mRNA levels of CXCL13 in ischemic kidneys were induced after 24 h also indicates that the source of CXCL13 production might be the damaged kidney. Although Tfhs are considered to be the major source of CXCL13, it has previously been shown that stromal cells can also produce CXCL13 in response to IL-17 in inflamed lung tissue in mice (23). Moreover, peripheral T helper cells, macrophages and damaged tubuli are potential candidate cells for CXCL13 production in kidney. Further studies are warranted to elucidate the underlying mechanisms of how CXCL13 expression is up-regulated in IRI and transplantation.

sc-RNAseq has emerged as a powerful technique for determination of different cell types (24, 25) and availability of new transcriptomic data expands the understanding of molecular mechanisms in various disease states. Accordingly, immune cell landscapes in particular renal disorders have been demonstrated (i.e., diabetic nephropathy, lupus nephritis) (26, 27). In the current study sc-RNAseq analysis was applied to identify cells that express the CXCL13 receptor CXCR5. It is shown that IRI alone caused substantial B-cell infiltration into the kidney within 7 days of IRI and a subset of B-cells was positive for CXCR5. The expression of CXCR5 was not limited to B-cells as a subset of renal T-cells and macrophages were also identified to be positive for CXCR5. Several lines of existing evidence points to a detrimental role for CXCR5+ cells in transplant rejection. Notably, in a recent study, the presence of CXCR5+ CD4+ cells correlated with acute rejection after liver transplantation (10). In ABMR, CXCR5 containing exosomes from Tfhs were significantly higher compared to controls and in co-culture experiments these Tfh derived exosomes were able to promote B-cell proliferation and maturation (28). Tfhs play a critical role in germinal center reactions and development of tertiary lymphoid structures which have been reported in chronic allograft rejection of kidney (29, 30), lung (31), and heart transplants (32). These germinal centers are the primary sites of B-cell expansion and maturation directing the production of antibodies (33). However, it needs to be pointed out that the findings in this study are correlational with regard to CXCL13 and CXCR5. Further studies using strategies to block CXCL13 are required to validate the hypothesis that infiltration of CXCR5 positive cells in ischemic kidneys is a direct result of CXCL13 secretion. Alternatively, it is feasible that the recruitment of these cells is due to ongoing renal inflammation, independent of CXCL13 secretion. Due to necrotic cell death in ischemic tissue a variety of damage-associated molecular patterns (DAMPs) are released which can interact with TLR2 and−4 expressed on myeloid, dendritic, tubular epithelial and endothelial cells. TLR activation mediates downstream production of TNF-α, IL-1ß, and IL-6 promoting activation of the adaptive immune response (34, 35). Based on the findings we propose that systemic expression of CXCL13 after IRI and DGF might be a potential clinical candidate for early detection of interstitial inflammation and B-cell activation.

Further clinical studies are needed to determine the role of CXCL13 as a predictive biomarker for AKI, DGF and rejection in ktx.

## Data Availability Statement

The datasets generated for this study are available on request to the corresponding author.

## Ethics Statement

The animal study was reviewed and approved by Lower Saxony State Office for Consumer Protection and Food Safety.

## Author Contributions

FG designed and supervised the experimental studies. FG and SI drafted the manuscript. KK, M-SJ, SR, VV, and LW conducted the experimental studies. SR performed IRI and ktx surgeries. FG and JB analyzed the histology and immunohistochemistry. WG, CK, LS, and HH discussed the results and edited the manuscript. SV, RS, and OD-B performed single cell sequencing experiments. All authors participated in the interpretation of data, editing, and approval of the manuscript.

## Conflict of Interest

The authors declare that the research was conducted in the absence of any commercial or financial relationships that could be construed as a potential conflict of interest. The handling editor declared a past co-authorship with one of the authors FG.

## Supplementary Material

Supplementary Figure 1 Clinical parameters in bilateral ischemia model. Fifteen minutes bilateral IRI did not cause relevant increase of s-creatinine **(A)** or BUN **(B)**. Bilateral IRI for 30 min caused significant deterioration of renal function with highly significant s-creatinine and BUN elevation (*n* = 5 sham mice, *n* = 6 mice per IRI group, one-way ANOVA, ^***^*p* < 0.001). BL, baseline.
