# Supplementary figures and images for: Ischemia Reperfusion Injury Triggers CXCL13 Release and B-Cell Recruitment After Allogenic Kidney Transplantation
Source: Front Immunol. 2020 Aug 6;11:1204. doi: 10.3389/fimmu.2020.01204 (PMC7424013; doi:10.3389/fimmu.2020.01204)

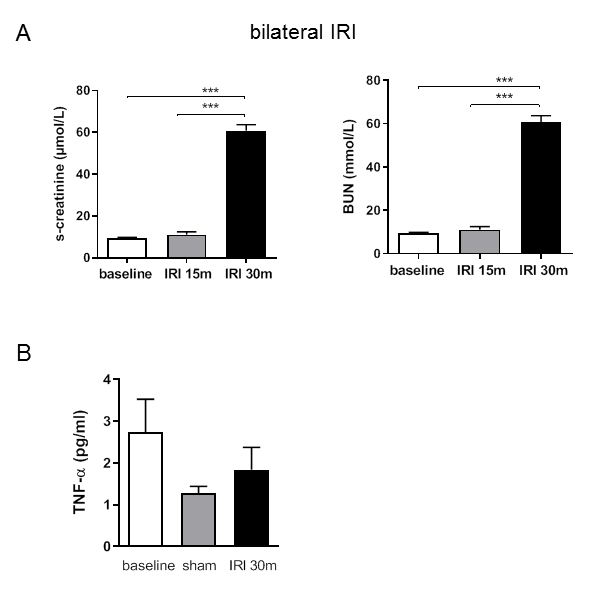

Supplement: Supplementary Figure 1 — Clinical parameters in bilateral ischemia model. Fifteen minutes bilateral IRI did not cause relevant increase of s-creatinine (A) or BUN (B). Bilateral IRI for 30 min caused significant deterioration of renal function with highly significant s-creatinine and BUN elevation (n = 5 sham mice, n = 6 mice per IRI group, one-way ANOVA, ***p < 0.001). BL, baseline. [file Image_1.TIF]
